# Ongoing Developments in Sporadic Inclusion Body Myositis

**DOI:** 10.1007/s11926-014-0477-9

**Published:** 2014-11-18

**Authors:** Pedro M. Machado, Mhoriam Ahmed, Stefen Brady, Qiang Gang, Estelle Healy, Jasper M. Morrow, Amanda C. Wallace, Liz Dewar, Gita Ramdharry, Matthew Parton, Janice L. Holton, Henry Houlden, Linda Greensmith, Michael G. Hanna

**Affiliations:** 1 MRC Centre for Neuromuscular Diseases, Institute of Neurology, University College London, Box 102, 8-11 Queen Square, London, WC1N 3BG UK; 2 Sobell Department of Motor Neuroscience and Movement Disorders, Institute of Neurology, University College London, Queen Square, London, WC1N 3BG UK

**Keywords:** Inclusion body myositis, Myositis, Myopathy, Neuromuscular, Genetics, Whole-exome sequencing, Aetiopathogenesis, Diagnosis, Treatment, Magnetic resonance imaging, Histology, Autoantibodies, Protein aggregates, Inflammation, Exercise, Disability, Myostatin, Monoclonal antibodies, Gene therapy, Heat shock proteins, Chaperones, Clinical trial

## Abstract

Sporadic inclusion body myositis (IBM) is an acquired muscle disorder associated with ageing, for which there is no effective treatment. Ongoing developments include: genetic studies that may provide insights regarding the pathogenesis of IBM, improved histopathological markers, the description of a new IBM autoantibody, scrutiny of the diagnostic utility of clinical features and biomarkers, the refinement of diagnostic criteria, the emerging use of MRI as a diagnostic and monitoring tool, and new pathogenic insights that have led to novel therapeutic approaches being trialled for IBM, including treatments with the objective of restoring protein homeostasis and myostatin blockers. The effect of exercise in IBM continues to be investigated. However, despite these ongoing developments, the aetiopathogenesis of IBM remains uncertain. A translational and multidisciplinary collaborative approach is critical to improve the diagnosis, treatment, and care of patients with IBM.

## Introduction

Sporadic inclusion body myositis (IBM) is an acquired muscle disease for which there is no effective treatment. IBM has a male predominance and usually does not affect individuals below the age of 45. The prevalence of the disease is in the range 1–71 per million, reaching 139 per million over the age of 50 [1–10]. Therefore, IBM belongs to the rare (or orphan) diseases, defined in the European Union as having a prevalence of no more than 5/10,000 people (500 per million) and defined in the United States of America as having a prevalence of fewer than 200,000 affected Americans (approximately 6.3/10,000 people or 630 per million). The rarity of the disease, lack of patient and clinical awareness, and diagnostic difficulties contribute to a substantial delay between the onset of symptoms and diagnosis (five-year delay on average) [11–13, 14•].

The aetiopathogenesis of IBM remains unclear. Historically it has been included in the group of idiopathic inflammatory myopathies, with polymyositis (PM), dermatomyositis (DM), and the immune-mediated necrotising myopathies. However, IBM is very different from these conditions and is characterized by lack of response to immunosuppressant medication, both inflammatory and degenerative features on muscle biopsy, a typical early clinical phenotype with (often asymmetric) weakness of the knee extensors and finger flexors, potential involvement of pharyngeal and upper-oesophageal muscles (which may contribute to malnutrition and aspiration), and progressive and slow deterioration that may lead to severe disability and loss of quality of life [12, 13, 14•, 15, 16].

In this article we will review ongoing developments in IBM, covering the genetic contribution to IBM, recent diagnostic developments, and the effect of exercise in IBM, and discuss new insights into the pathogenesis of the disease and new therapeutic approaches, with a focus on targeting protein dyshomeostasis and inhibiting the myostatin pathway.

## Genetic Contribution to IBM

Although it is regarded as a sporadic disease, genetic studies are important in IBM because they may reveal important pathways involved in the disease and risk factors that may increase our understanding of this disorder and identify potential therapeutic targets. There are three genetic approaches best suited to studying IBM:

1. candidate gene analysis, on the basis of clinico-pathological features;
2. genetic analysis of small families with IBM or very similar clinical and pathological phenotypes; and
3. large-scale genome-wide association study (GWAS) and exome-sequencing approaches [17••].

### Candidate Gene Studies Have Provided Evidence of Genetic Susceptibility in IBM

Major histocompatibility complex (MHC) associations still provide the strongest evidence for a genetic component to IBM. The strong association of the extended 8.1 ancestral haplotype (AH) with IBM has been reported in a series of studies on different Caucasian populations [18–26]. Other alleles and haplotypes have also been associated with increased risk of IBM, including 35.2AH [22] and 52.1AH [27] for Caucasians and Japanese populations, respectively. Furthermore, two polymorphisms (rs422951 and rs72555375) of the *NOTCH4* gene in a susceptibility region for IBM with MHC (from *BTNL2* to *HLA-DRB1*) [28] also had a strong association (OR >2) with IBM in two independent Caucasian cohorts [29]. Further investigations are required to determine whether these genes are directly involved in the disease pathogenesis. Recently, an autoantibody against the cytoplasmic 5′-nucleotidase 1A (cN1A; *NT5C1A*) was identified in IBM [30••, 31••]. Although no protein-coding mutation was found in a small group of patients, *NT5C1A* and its related genes remain important candidate genes for future investigation.

Research on candidate genes has also focused on the genes encoding the aggregated proteins or proteins related to neurodegenerative disease. Studies have included, for example, beta-amyloid precursor protein (*APP*) [32, 33], apolipoprotein E (*APOE*) [34], phosphorylated Tau (*MAPT*) (unpublished data), alpha-1-antichymotyrpsin (*SERPINA3*) [35], prion protein (*PRNP*) [36–38], TAR DNA-binding protein-43 (TDP-43; *TARDBP*) [39–41], and *C9orf72* (unpublished data). However, no associations between these genes and IBM have yet been established.

Mutations in mitochondrial DNA (mtDNA) have also been investigated. Multiple mtDNA deletions have been reported in many cytochrome-c-oxidase (COX)-deficient ragged-red fibres of IBM patients [42–44]. Mutations of mitochondrial encoded nuclear genes, including *TYMP*, *SLC25A4* (previously known as *ANT1*), *C10orf2*, and *POLG1*, had previously been associated with multiple mtDNA deletions, and therefore these genes were studied; however, no mutations were found in five IBM patients [45]. Interestingly, an intronic polymorphism (rs10527454, described as ‘rs10524523’ in currently published papers) in a gene called “translocase of outer mitochondrial membrane 40” (*TOMM40*) which is adjacent to and in linkage disequilibrium with the *APOE* locus on chromosome 19 [46], together with the *APOE* genotypes, has been revealed to affect IBM disease susceptibility [47].

### Genes Identified in Familial or Hereditary IBM, and Other Vacuolar and IBM-Like Myopathies, Also Provide Important Insights for IBM Genetic Research

Table 1 summarises the genes identified in familial or hereditary IBM that may provide insight for IBM genetic research [17••, 48–50]. In a Japanese study, the p.V805A variant in myosin heavy chain IIa (*MYH2*) (gene associated with hIBM3) significantly increased the risk of developing IBM (RR =12.2) in a group of 21 patients [51].

**Table 1:** Genes identified in familial or hereditary IBM that may provide insights for IBM genetic research

IBM-like diseases	Genes
Familial IBM	*HLA*-*DR3* allele (DRB*0301/0302); *HLA*-*DR15*(2)/*DR4* (DRB1*1502/0405)
Hereditary IBM	*GNE* (OMIM#603824); *LAMA2* (OMIM#156225); *MYH2* (OMIM#160740); *VCP* (OMIM#601023)
Other rimmed vacuolar myopathies	*PABPN1* (OMIM#602279); *EMD* (OMIM#300384); *MYOT* (OMIM#604103); *TCAP* (OMIM#604488); *SEPN1* (OMIM#606210)

### Exome, Genome, and GWAS Approaches

Although IBM is not an inherited Mendelian disease, multiple genetic risk factors have been proposed to have important functions in the development and progression of IBM. The advent of more robust genetic approaches, for example whole-exome sequencing, has enabled the identification of rare coding variants which may be functional, increasing the probability of detecting disease-associated variants. This is particularly important for such rare diseases as IBM, where the number of cases is probably not large enough for a conventional GWAS. Our group is currently collecting IBM samples in an exome-sequencing project to perform an approach to identify novel risk pathways. We have exome sequenced more than 100 IBM cases and we hope to increase this number through collaboration with neurologists and rheumatologists around the world.

## Histopathological Findings and Their Diagnostic Use

In addition to the inflammatory changes observed, muscle biopsy in IBM reveals a wide range of pathological features including variation in fibre size, rounded and angulated atrophic fibres, increased numbers of internal nuclei, mitochondrial changes including COX-negative fibres and ragged-red fibres, and increased endomysial connective tissue.

Historically, diagnostic criteria for IBM have depended heavily upon the observation of specific pathological findings on muscle biopsy. The seminal Griggs criteria were the first widely adopted diagnostic criteria for IBM [52]. Using these criteria, a diagnosis of definite IBM could be made solely on the basis of the following pathological findings: an auto-aggressive inflammatory myopathy with invasion of morphologically normal fibres (so called partial invasion), rimmed vacuoles (irregular vacuoles within a muscle fibre surrounded by or containing basophilic granular material with haematoxylin and eosin staining, or staining red with Gomori trichrome), and either amyloid or 15–18 nm tubulofilamentous inclusions visualised with electron microscopy (EM). These pathological findings, in isolation, are found in other myopathies; however, in combination they are regarded as highly specific for IBM. The recognition of the characteristic clinical presentation associated with IBM has revealed that these diagnostic pathological features may be absent in patients with clinically typical IBM [14•, 53]. One study found that more than 40 % of patients lacked the necessary diagnostic pathological features on light microscopy at presentation, and lent support to the theory that the limited sensitivity of the pathological features included in the Griggs criteria is because they are associated with chronologically more advanced disease [54].

Immunohistochemical staining techniques have clarified the composition of the inflammatory infiltrate in IBM [55], revealed the widespread sarcolemma and sarcoplasmic upregulation of MHC class I (MHC-I) [56••], and identified the pathological accumulation of many proteins within muscle fibres in IBM. The proteins most frequently described include: proteins more commonly associated with neurodegenerative diseases, namely β-amyloid, phosphorylated tau, and ubiquitin; the myofibrillar-myopathy-associated proteins myotilin and αB-crystallin; and the newer neurodegenerative markers p62 and TDP-43. However, some of these findings have not been consistently reproduced, leading to uncertainty and questions over their significance [57].

Despite a lack of data on the diagnostic utility of the pathological findings, most expert muscle pathologists agree that histochemical staining for mitochondrial changes and immunohistochemical staining aid in differentiating IBM from pathologically similar myopathies. Two recent quantitative studies examined the sensitivity and specificity of pathological features in IBM. The first recommended a combination of LC3 and TDP-43 staining: <14 % LC3-positive fibres helped to exclude a diagnosis of IBM, and >7 % TDP-43-positive fibres supported a diagnosis of IBM [58•]. The main limitation of this study was a lack of clinical data. A subsequent study assessed the diagnostic utility of markers of protein aggregation, inflammation, and mitochondrial changes [56••]. The authors proposed a pathological diagnostic algorithm to differentiate IBM with rimmed vacuoles from other vacuolated myopathies (sensitivity 93 %, specificity 100 %) and IBM without rimmed vacuoles from steroid-responsive inflammatory myopathies (sensitivity 100 %, specificity 73 %). An additional finding was that the morphology of p62 aggregates in IBM was consistent and may aid in differentiating IBM from pathological mimics.

## Autoantibodies

Autoantibodies against cN1A are a new serological marker for IBM [30••, 31••, 59]. With their high specificity and moderate sensitivity for IBM, anti-cN1A antibodies may be particularly useful in the differential diagnosis of recent-onset myopathies and/or myositis. Interestingly, the anti-cN1A response has been revealed to consist of a variety of immunoglobulin classes, and this could also be of diagnostic value [60]. Using absorbance units (AU) to measure anti-cN1A reactivity, an IgG cut-off >0.9 AU had 51 % sensitivity and 94 % specificity, an IgA cut-off >1.2 AU had 49 % sensitivity and 95 % specificity, and an IgM cut-off >1.9 AU had 53 % sensitivity and 96 % specificity for diagnosing IBM in a population of 205 individuals: 50 with IBM, 121 with another neuromuscular disease, and 34 healthy controls. By testing several thresholds and combining the three isotypes, it was possible to increase sensitivity to 76 %, with only a slight reduction in specificity (91 %), using the following combination of cut-offs: IgG >1.3 AU or IgA >1.1 AU or IgM >1.9 AU [60].

## MRI Assessment

**Fig. 1:**
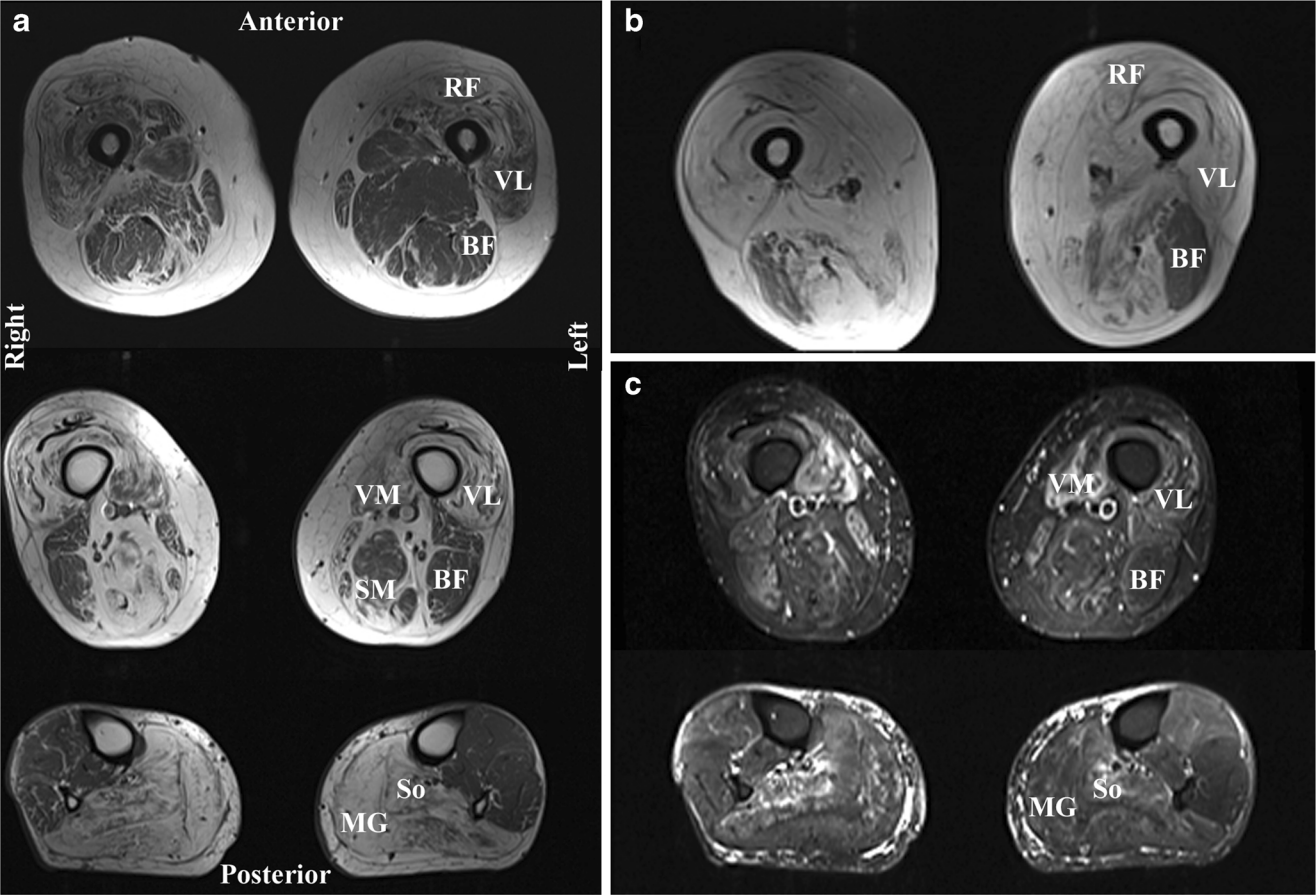
Typical MRI appearances in a patient with IBM. (**a**) Axial T1-weighted images of the mid-thigh (top), distal thigh (middle), and mid-calf (bottom). The thigh shows intramuscular fat accumulation, evident as hyperintensity; most notably within the quadriceps (RF: rectus femoris; VL: vastus lateralis; VM: vastus medialis), especially in the distal thigh. Hamstring involvement is asymmetric, with semimembranosus (SM) relatively spared on the left. In the calf the medial gastrocnemius (MG) is completely replaced by fat, with the soleus (So) also severely affected. (**b**) Axial T1-weighted image at mid-thigh of the same patient six years later shows significant progression of intramuscular fat accumulation, with only the biceps femoris (BF) relatively unaffected. (**c**) Axial STIR images at distal thigh (top) and mid-calf (calf) in the same patient at baseline. Acute muscle inflammation is evident as hyperintensity, most markedly in the vastus medialis and soleus

Muscle MRI is increasingly recognised as a useful assessment in the diagnostic pathway of inherited muscle disease [61]. In IBM patients both signal hyperintensity within muscles on T1-weighted sequences, caused by intramuscular fat accumulation, and hyperintensity caused by muscle oedema on T2-weighted sequences with fat suppression, for example the short tau inversion recovery (STIR) sequence, are observed (Fig. 1). A selective pattern of muscle involvement has been reported. Similar to the clinical presentation, within the forearm there is preferential intramuscular fat accumulation within the flexor digitorum profundus [62, 63•, 64, 65]; whereas in the thigh the quadriceps femoris is preferentially affected [63•, 64, 66], with some authors reporting relative preservation within the quadriceps of the rectus femoris [63•, 64]. Within the lower leg, the medial head of the gastrocnemius consistently has maximal intramuscular fat accumulation [63•, 64, 66]; a feature not apparent clinically as ankle plantar flexion weakness, because the soleus and the lateral gastrocnemius have lesser involvement. STIR hyperintensity reflecting active inflammation within the muscle is commonly seen, but in a smaller number of muscles than are affected by fat accumulation [62, 63•, 65]. Although this typical pattern of involvement is well described, the diagnostic sensitivity and specificity of MRI in IBM has not been systematically assessed and MRI appearances are not currently included in IBM diagnostic criteria.

In addition to this potential function in the diagnosis of muscle disease, MRI shows much promise as a tool to monitor disease progression and provide sensitive outcome measures for clinical trials. MRI is able to quantify intramuscular fat accumulation with the three-point Dixon sequence [67] most commonly used. Acute pathology can be quantified by measuring increases in T2 within muscle; these may be reversible with effective therapy, as revealed for periodic paralysis [68]. These quantitative MRI methods have been revealed to have good reliability for healthy volunteers [69]. In IBM patients, intramuscular fat accumulation within the quadriceps has a strong negative correlation to knee-extension strength [70]. Unlike strength testing, MRI is independent of subject effort, so may provide more responsive measures of disease progression than direct myometric assessment. Performance of MRI outcome measures should be defined in IBM natural-history studies before application in clinical trials.

## Research Diagnostic Criteria

The 2011 European Neuromuscular Centre (ENMC) criteria were published in 2013 and are the latest IBM research diagnostic criteria [71•]. They build on the previously published MRC Centre criteria [72, 73]. In the presence of the appropriate clinical phenotype, the ENMC criteria enable more flexibility regarding the presence of typical histopathological features. Patients can be divided into three categories: “clinico-pathologically defined IBM”, “clinically defined IBM”, and “probable IBM” (Table 2) [71•].

**Table 2:** 2011 European Neuromuscular Centre Inclusion Body Myositis research diagnostic criteria 2011 (adapted from Ref. [71•])

Category	Clinical features	Pathological features
Clinico-pathologically defined IBM	○ Duration of weakness >12 months ○ Creatine kinase ≤15 × ULN ○ Age at onset >45 years ○ FF weakness > SA weakness *and*/*or* KE weakness ≥ HF weakness	*All of the following*: ○ Endomysial inflammatory infiltrate ○ Rimmed vacuoles ○ Protein accumulation^a^ or 15–18 nm filaments
Clinically defined IBM	○ Duration of weakness >12 months ○ Creatine kinase ≤15 × ULN ○ Age at onset >45 years ○ FF weakness > SA weakness *and* KE weakness ≥ HF weakness	*One or more, but not all, of:* ○ Endomysial inflammatory infiltrate ○ Up-regulation of MHC class I ○ Rimmed vacuoles ○ Protein accumulation^a^ or 15–18 nm filaments
Probable IBM	○ Duration of weakness >12 months ○ Creatine kinase ≤15 × ULN ○ Age at onset >45 years ○ FF weakness > SA weakness *or* KE weakness ≥ HF weakness	*One or more, but not all, of*: ○ Endomysial inflammatory infiltrate ○ Up-regulation of MHC class I ○ Rimmed vacuoles ○ Protein accumulation^a^ or 15–18 nm filaments

^a^Evidence of amyloid or other protein accumulation by established methods (e.g. for amyloid Congo red, crystal violet, thioflavin T/S, for other proteins p62, SMI-31, TDP-43)

FF, finger flexion; HF, hip flexion; KE, knee extension; SA, shoulder abduction; MHC class I, major histocompatibility complex class I; ULN, upper limit of normal

In a retrospective population of 371 patients (200 with and 171 without IBM), specificity was high (98–100 %) for all three categories. The sensitivity was 15 % for “clinico-pathologically defined IBM” and 84 % for the combination of “clinically defined and probable IBM” [74••]. The overall sensitivity of the criteria was not reported, but given that patients with “clinico-pathologically defined IBM” may or may not satisfy the other two categories, the overall sensitivity of the criteria should be approximately 90 % (exact value in the range 84–99 %). In another study that included 67 patients with IBM, the reported sensitivity of the 2011 ENMC criteria was 88 % [54]. Therefore the 2011 ENMC criteria seem suitable for selecting patients for clinical trials, particularly if they are used as a whole (i.e. including all three categories). Individually, some IBM features perform better than others, and in the above-mentioned dataset of 371 patients the authors applied a machine-learning technique to IBM features and found that a simplified combination of finger-flexion or knee-extension weakness, endomysial inflammation, and either partial invasion or rimmed vacuoles had 90 % sensitivity and 96 % specificity for IBM [74••].

## Aetiopathogenesis

The aetiopathogenesis of IBM is controversial and probably multifactorial [75–78]. Several pathogenic models have been proposed, including autoimmunity, protein dyshomeostasis, myonuclear degeneration, nucleic-acid-metabolism impairment, mitochondrial dysfunction, and a function for microRNA and the myostatin pathway [75–86]. Ageing, genetic, and environmental factors may also contribute to disease aetiopathogenesis.[17••, 78] In this review we will focus on two models with recent therapeutic implications: protein dyshomeostasis and myostatin signalling.

### Protein Dyshomeostasis: Function in IBM

Mounting evidence suggests that there is disruption of protein homeostasis in IBM muscle, resulting from impaired protein degradation by autophagy and the ubiquitin–proteasome system (UPS), which may be responsible for the degenerative pathology observed in affected muscles.

#### Disruption of the UPS in IBM Muscle

Ubiquitinated proteins are catabolised by the proteasome in the UPS, the main cellular-protein degradation pathway. In IBM muscle, two proteins which target ubiquitinated proteins for degradation, valosin-containing protein (VCP) and p62, are aggregated in the sarcoplasm [87–90]. Heightened ubiquitination of proteins and a threefold increase in p62 expression in IBM muscle suggest either an increased need for protein degradation or impaired UPS or autophagy [90, 91]. Proteasome subunits have also been identified in sarcoplasmic aggregates [92], and others report a significant reduction in proteasome function at all of its three catalytic sites [90, 92]. Moreover, immuno-proteasome subunits, which produce peptides for MHC class I antigen presentation, have also been detected in affected IBM muscle, linking protein degradation to inflammation [92]. UBB^+1^, a mutant form of ubiquitin, is also expressed in IBM muscle [93], and high levels of UBB^+1^ may result in proteasome inhibition [93].

#### Disruption to Autophagy in IBM Muscle

Rimmed vacuoles, a defining characteristic of IBM, have been associated with abnormal lysosomal activity caused by the presence of membranous debris within the vacuoles, and an increased immuno-reactivity for markers of autophagy [90]. Autophagy components including LAMP2A and LC3 II, the autophagosome maturation marker, accumulate in IBM myofibres, indicating either an increased demand for autophagy or an impaired pathway [90, 94, 95]. Askanas and colleagues have identified a 50 % reduction in activity of lysosomal cathepsin D despite an increased protein load [90]. Furthermore, they reveal that levels of NBR1, an autophagy-associated ubiquitin-binding protein, are increased in IBM-patient muscle, with the protein aggregated in the sarcoplasm [96]. This indicates a lack of the functioning components crucial for efficient autophagy.

#### The Unfolded-Protein Response (UPR)

The expression levels of five proteins of the unfolded-protein response (UPR), an acute cellular response to misfolded proteins in the endoplasmic reticulum (ER) (causing ER stress), are elevated in IBM muscle [97]. Furthermore, aggregation of VCP, which extracts and transports ER proteins to the proteasome, suggests a broken link between the UPR and proteasomes in IBM [90, 98].

#### Evidence of Protein Mishandling in IBM

Disruption to the three protein-handling mechanisms described above leads to reduced protein clearance, resulting in protein accumulation and aggregation. The presence of inclusion bodies, large sarcoplasmic filamentous aggregates, is characteristic of IBM, and more than 50 different proteins have been associated with these aggregates, including TDP-43, presenilin1, amyloid β-precursor protein, and phosphorylated tau [90]. No single protein has been universally identified in all IBM muscle or been linked to pathogenesis, suggesting aggregation is non-specific [91]. However, it is possible that protein aggregation is not simply a diffusion-limited process, but rather a regulated tool to sequester excessive cellular proteins, thereby attempting to redress the imbalance in protein homeostasis [99].

Protein chaperones, for example heat-shock protein 70 (HSP70), bind to aberrant proteins and help prevent aggregation [100]. An increase in HSP70 expression, and HSP70 and αB-crystallin immuno-reactivity in IBM inclusion bodies [90], suggests a diminished capacity of chaperone proteins to handle the excessive protein load in IBM myofibres. These findings suggest that approaches with the objective of restoring protein homeostasis may be a successful therapeutic strategy for IBM.

### The Myostatin Pathway: Function in IBM

The myostatin pathway is a central negative regulator of myogenesis during development and periods of muscle regeneration in postnatal life [101]. Inhibition of this pathway enhances muscle regeneration in animal models of muscle disease [16]. Myostatin knockout mice also have an increase in muscle mass without organ anomalies [102], and a range of myostatin-inhibited animals, and a human patient with loss-of-function mutations, have muscle hypertrophy with increased strength [103–106]. Thus this pathway is of major interest as a target for therapeutic manipulation in neuromuscular conditions characterised by progressive muscle atrophy and weakness.

After activation, the myostatin peptide of the transforming-growth-factor-β superfamily binds to the transmembrane activin receptor IIB (ActRIIB), which in turn activates a Smad complex that enters the nucleus and activates the transcription of myogenic genes, inhibiting the proliferation and differentiation of myogenic precursors [107]. During development, myostatin inhibitors (follistatin, FLRG, and GASP-1) bind extracellularly, reducing bioavailability [16]. Antibodies to myostatin [108, 109], a propeptide to maintain its inactive state [110], a dominant negative myostatin analogue [111], and soluble ActRIIB [112] have all been designed to block the myostatin pathway, in addition to using isoforms of follistatin [113], follistatin gene therapy [113, 114], and the use of histone deacetylase inhibitors to up-regulate follistatin [115, 116].

Myostatin may be implicated in the pathogenesis of IBM, as suggested by Wójcik et al. in a study revealing the accumulation of myostatin in muscle fibres [117]. Myostatin signalling also leads to FOXO/Atrogin-1 induction [118], mediating atrophy by targeting the myogenic regulatory factor myoD for degeneration [119]. Nuclear translocation of the forkhead-family transcription factor Foxo3A and mRNA induction of the atrophy-related ubiquitin ligase Atrogin-1 have been revealed in IBM and polymyositis, indicating activation of this pathway [120]. Nonetheless, the central pathogenesis remains to be fully elucidated in IBM, and targeting this pathway would not specifically address the known degenerative and inflammatory and/or immune factors.

For example, enhancing regeneration without suppressing dysimmune factors could be counterproductive, as suggested by the potential to elicit autoantibodies to antigens enriched in regenerating fibres [121]. Furthermore, dysregulation of the autophaglyosome in IBM [122] may have consequences for the simultaneous therapeutic promotion of muscle mass, because the effective synthesis of structural proteins in aging patients would be essential to long-term treatment benefits. Also, an underlying problem with endogenous cellular-protective mechanisms (accumulation of misfolded proteins, endoplasmic-reticulum stress) [97] could compromise the therapeutic efficacy of myostatin inhibitors.

Although it has been argued that addressing the underlying pathogenic process is critical to designing therapy for IBM [16], promoting skeletal-muscle growth via myostatin inhibition may still be effective. This could reduce disability for patients during their lifetime, because IBM is a slowly progressing disease and potential clinical benefits may outpace the rate of functional decline.

## IBM Clinical Trials: Targeting Protein Dyshomeostasis and Inhibiting the Myostatin Pathway

In a recent randomised double-blind placebo-controlled trial (RDBPCT) [123, 124], up-regulation of the heat-shock response (HSR) was tested by treatment with arimoclomol, an oral pharmacological agent that has been revealed to co-induce the synthesis of heat-shock proteins (including HSP70 and HSP90) by augmenting the HSR in cells where the endogenous HSR has already been initiated.

In this clinical trial, 24 patients were randomised with a 2:1 arimoclomol–placebo ratio. The investigational drug was administred for four months and arimoclomol was given at a dose of 100 mg TDS. After a four-month treatment phase there was an additional eight-month blinded follow-up phase. No major safety problems were observed during the trial, and the drug was well tolerated by patients. Efficacy measures were secondary outcomes. Numerically, the rate of decline in physical function (IBM functional rating scale) and muscle strength (right-hand-grip maximum voluntary isometric contraction testing and manual muscle testing) was less in the arimoclomol group compared with placebo, with a stronger (non-statistically significant) trend at eight months. This preliminary indication of the potential therapeutic benefit of arimoclomol supports further investigation of this drug for treating patients with IBM.

Two myostatin antagonists are currently being tested for IBM. The first approach uses follistatin gene therapy (FS344) delivered by adeno-associated virus (AAV) and administered by quadriceps intramuscular injection [125]. This open-label clinical trial will test the safety of three different doses of AAV-FS344 (total nine patients, three patients per dose-treatment group). Measures of muscle strength, physical function, thigh circumference, MRI assessment, and muscle histopathology are secondary outcomes of the study [125].

The second anti-myostatin approach uses a humanised monoclonal antibody (BYM338/Bimagrumab) that binds to ActRIIB and therefore blocks the effect of myostatin. The drug is administered intravenously and was recently tested in a small single high-dose pilot study [109, 126]. After eight weeks of drug administration, thigh-muscle volume increased in the BYM338 group (11 patients) versus placebo (3 patients) (6.5 % and 7.6 % increase in the right and left thigh, respectively), as did fat-free mass (5.7 % increase). After 16 weeks of follow-up (with two drop-outs), patients who had been given the active drug had a 14.6 % increase in their 6 min walking distance (6MWD) compared with those given placebo. All these differences were statistically significant [126].

Bimagrumab is now being tested in a large multicentre RDBPCT that intends to recruit 240 patients throughout the world [127]. This phase IIb and III clinical trial will test three different monthly doses of the active drug versus placebo (1:1:1:1 ratio). This will be the largest IBM clinical trial to date and will last at least one year. The primary outcome measure is the 6MWD. Several secondary outcome measures will be evaluated, including muscle strength, lean body mass, disability, health-related quality of life, rate of fall events, swallowing function, and safety and tolerability of the drug [127].

## Exercise in IBM

Lack of restorative treatment for IBM means that exercise has an important function in managing symptoms and reducing physical inactivity with its associated sequelae [128, 129•]. Although historically people with muscle conditions were advised not to exercise for fear of increasing damage, in recent years several small non-randomised studies have revealed benefits of exercise in neuromuscular conditions [128, 130].

For people with IBM, moderate-intensity strength training is established as safe (by serum markers of inflammation and muscle biopsy), with mixed reports on efficacy [131–133]. However, these studies consisted of small cohorts, were heterogeneous with respect to disability, and lacked controls and blinding.

Improved quadriceps muscle strength could potentially have a positive effect on disability, because it has been revealed to correlate with improved walking and stair-climbing tests [134]. New approaches also show promise, including a recent case study reporting increased strength with resistance training and vascular occlusion for someone who had previously reached a plateau [135]. However, quadriceps strength as a surrogate marker has yet to be validated, and good quality randomised controlled trials are needed to determine the best type, dose, and intensity of training.

Aerobic deconditioning is highly prevalent in people with neuromuscular conditions, and is a consequence of reduced general activity levels [136–138]. Aerobic training has not been rigorously examined for people with IBM, although one study did test a combined strength and aerobic home-training programme [139]. Participants cycled three times a week at 80 % maximum heart rate, with strength training performed on alternate days. However, details in the paper are limited; it seems that intensity and dose were low, with participants cycling for short periods, and with strength training at a reduced frequency compared with previous studies [133] and with low load. The authors revealed increased cardiorespiratory fitness, but measured this using a submaximal exercise test which has known reliability problems [140]. Recently there has been much interest in high-intensity interval training as a way to improve fitness without increasing fatigue. One group has started to test this for IBM [141], but full results are not yet available and safety is yet to be established.

Other potential exercise interventions have not been investigated for IBM; for example, specific balance exercises for falls prevention or exercises for weak upper-limb, hand, and trunk muscles, all of which have a strong effect on functional ability and quality of life. Timing of intervention has also not been systematically tested—could strength training early in the disease process slow or reduce functional impairment in the later stages?

Unfortunately, increasing evidence for the safety and efficacy of exercise has not reached the patient population. A recent study found they spent less time exercising and reported more barriers to exercise than controls. The most common barriers were lack of energy and motivation, and concerns about health, pain, and accelerating the degenerative process [142]. Good-quality evidence is needed so people with IBM can be confident their exercise prescription will lead to functional improvement with minimum adverse effects.

Our group is investigating aerobic training in IBM regarding fitness levels, muscle strength, and function, using a randomised crossover design with training and control periods [143]. Training consists of cycling three times a week for twelve weeks at 60 to 80 % of heart-rate reserve. The primary outcome measure is VO_2_ peak, with strength and function as secondary outcomes. Aspects of the intervention including engagement, motivation, psychological well-being, and the patient experience will also be investigated, because these are crucial for the optimum uptake of exercise recommendations.

## Conclusion

These are exciting times for research in IBM. A large amount of work has been undertaken in recent years to improve our understanding of IBM and to enhance our capacity to optimally diagnose and monitor the disease. Despite recent advances, the precise aetiology of the disease remains unknown and the mechanisms of interaction between the different pathogenic models that have been proposed remain to be clarified. There is no pharmacological treatment that has proved to be *efficacious in* IBM clinical trials. However, the prospect is still encouraging, with ongoing research developments and findings from pre-clinical experiments being translated into new clinical trials. IBM is a rare disease, and the development of global strategies capable of fostering research collaboration will be crucial to improve diagnosis, treatment, and care for people with this debilitating disease.
